# Effects and molecular mechanisms of intrauterine infection/inflammation on lung development

**DOI:** 10.1186/s12931-018-0787-y

**Published:** 2018-05-10

**Authors:** Jiarong Pan, Canyang Zhan, Tianming Yuan, Weiyan Wang, Ying Shen, Yi Sun, Tai Wu, Weizhong Gu, Lihua Chen, Huimin Yu

**Affiliations:** 1 Department of Neonatology, Children’s Hospital, Zhejiang University School of Medicine, 3333 Binsheng Road, Hangzhou, Zhejiang, 310052 People’s Republic of China; 2 Zhejiang Key Laboratory for Diagnosis and Therapy of Neonatal Disease, 3333 Binsheng Road, Hangzhou, Zhejiang, 310052 People’s Republic of China

**Keywords:** Intrauterine infection, Inflammation, Bronchopulmonary dysplasia, Expression profiling, MicroRNA

## Abstract

**Background:**

Intrauterine infection/inflammation plays an important role in the development of lung injury and bronchopulmonary dysplasia (BPD) in preterm infants, While a multifactorial genesis is likely, mechanisms involved in BPD after intrauterine infection/inflammation are largely unknown. Recent studies have suggested microRNAs (miRNAs) are likely to play a role. Therefore, this study aimed to study the effects and mechanisms of intrauterine infection/inflammation on lung development, and to identify miRNAs related to lung injury and BPD.

**Methods:**

An animal model of intrauterine infection/inflammation was established with pregnant SD rats endocervically inoculated with E.coli. The fetal and neonatal rats were observed at embryonic day (E) 17, 19, 21 and postnatal day (P) 1, 3, 7, 14, respectively. Body weight, lung weight, the expression levels of NLRP3, TNF-α, IL-lβ, IL-6, VEGF, Collagen I, SP-A, SP-B and SP-C in the lung tissues of fetal and neonatal rats were measured. Expression profiles of 1218 kinds of miRNAs in the lungs of neonatal rats were detected by miRNA microarray technique. Target genes of the identified miRNAs were predicted through online software.

**Results:**

Intrauterine infection/inflammation compromised not only weight development but also lung development of the fetal and neonatal rats. The results showed significantly increased expression of NLRP3, TNF-α, IL-1β, IL-6, Collagen I, and significantly decreased expression of VEGF, SP-A, SP-B and SP-C in the fetal and neonatal rat lung tissues in intrauterine infection group compared to the control group at different observation time point (*P* < 0.05). Forty-three miRNAs with significant differential expression were identified. Possible target genes regulated by the identified miRNAs are very rich.

**Conclusions:**

Intrauterine infection/inflammation results in lung histological changes which are very similar to those observed in BPD. Possible mechanisms may include NLRP3 inflammasome activation followed by inflammatory cytokines expression up-regulated, inhibiting the expression of pulmonary surfactant proteins, interfering with lung interstitial development. There are many identified miRNAs which target a wide range of genes and may play an important role in the processes of lung injury and BPD.

**Electronic supplementary material:**

The online version of this article (10.1186/s12931-018-0787-y) contains supplementary material, which is available to authorized users.

## Background

**Fig. 1:**
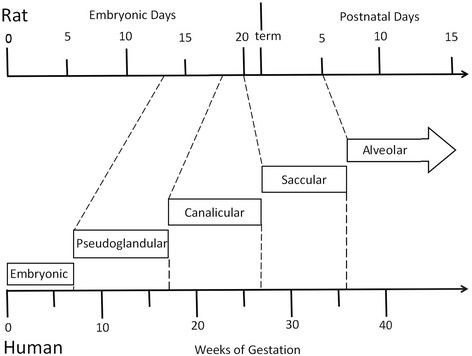
Schematic diagram depicting stages and gestational ages of lung development in humans and rats. The period of lung development has been divided into five stages: embryonic stage, pseudoglandular stage, canalicular stage, saccular stage and alveolar stage in humans and rats

The period of antenatal development of the human lung has been divided into five stages: embryonic stage, pseudoglandular stage, canalicular stage, saccular stage and alveolar stage (Fig. 1). Bronchopulmonary dysplasia (BPD), a common pulmonary complication mainly seen in premature infants born at the canalicular and saccular stages, shows a multifactorial pathogenesis. Intrauterine infection can lead to intrauterine inflammation which not only triggers premature birth as an important independent risk factor but also plays an important role in the development of BPD [1, 2]. However, the precise molecular biological mechanisms of intrauterine infection/inflammation on fetal and neonatal lung development are unclear.

The NLRP3 (NOD-like receptor family, pyrin domain containing 3) inflammasome is a protein complex that orchestrates immune responses to infection and cell stress through activation of caspase-1 and inflammatory cytokines [3]. Activation of the NLRP3 inflammasome during infection can be protective, but unregulated NLRP3 inflammasome activation in response to endogenous or exogenous stimuli can result in pathologic damage. Some researchers conclude that early activation of the NLRP3 inflammasome is a key mechanism in the development of BPD [4].

MicroRNAs (miRNAs) are short non-coding RNA molecules with a length of approximately 22 nucleotides that are involved in RNA silencing and post-transcriptional regulation of gene expression. Some recent studies suggested miRNAs participate in the processes of respiratory development, infection, inflammation, fibrosis and tumorigenesis [5–8]. However, whether miRNAs are operative in BPD, either in animal models of the disease or in infants with BPD, is unknown. The research of the characteristics of miRNAs expression in developmental phases of lungs with injury after intrauterine infection/inflammation has not been reported yet.

We utilised pregnant Sprague-Dawley (SD) rats endocervically inoculated with E.coli suspension to investigate the effects of intrauterine infection/inflammation on lung development at different time in fetal rats and neonatal rats. We hypothesized that intrauterine infection/inflammation could be associated with the development of BPD and cause the changes of miRNAs which might be important factors for adverse lung development.

## Methods

### Animals

All procedures involving animals were performed at Zhejiang University (Hangzhou, Zhejiang, People’s Republic of China) following review and approval by the Laboratory Animal Welfare and Ethics Committee of Zhejiang University. Twenty four date-mated SD rats with pregnancies were anaesthetized with an intraperitoneal dose of 40 mg/kg body weight of 2% sodium pentobarbital at embryonic day15 (E15) and assigned at random to receive either: i) two vaginal dilator-guided intramuscular injections of 0.2 mL saline on both the left and right sides of cervix (control group, *n* = 12); or ii) two vaginal dilator-guided intramuscular injections of 0.2 mL E.coli suspension on both the left and right sides of cervix (intrauterine infection group, n = 12). Successful placement of endocervically intramuscular injections was confirmed with histological examination of the placenta and uterus using a CX-21DIN Microscope (Olympus, Japan). No pregnant rats death occurred in the E.coli or Saline control groups.

Seven SD rats with pregnancies from each group were anaesthetized with intraperitoneal sodium pentobarbital (40 mg/kg), and fetuses were surgically delivered at E17, E19 and E21. The fetal and neonatal rats were observed and sacrificed by breaking cervical vertebrae immediately after being weighed at E17, E19, E21and postnatal day (P) 1, 3, 7, 14, respectively. Body weight, lung weight and lung/body weight ratio of 6 pups from each group were measured at every observation time point. Fetal and neonatal lung tissues were removed and processed as follows: the right upper lobe of lung was fixed in neutral buffered formalin for histological examination; the other lobes were snap frozen and kept at − 80 °C for protein, mRNA, and miRNA expression analyses.

### E.Coli suspension for endocervically intramuscular injection

A strain of E.coli (E.coli ATCC-25922 supplied by bacteriological laboratory of Children’s Hospital, Zhejiang University School of Medicine) was cultured on MacConkey agar at 37 °C for 24 h and single colonies were inoculated into sterile saline. Inoculums were quantified using a plate dilution series as standard microbiological methods and E.coli suspension was prepared at a concentration of 1MCF (2.5–3.5 × 10^8^ CFU/mL) prior to use.

### Histology

Four μm-thick sections from neutral buffered formalin fixed lung (right upper lobe), placenta or uterine tissues embedded in paraffin blocks were stained with haematoxylin and eosin.

### Immunohistochemical staining and scoring

Lung samples were obtained and treated as previously described. In addition, sections to be stained for NLRP3, vascular endothelial growth factor (VEGF) and Collagen I were deparaffinized and dehydrated with xylene and graded alcohols. Antigen retrieval was performed. Endogenous peroxidase activity was quenched by incubation with 3% hydrogen peroxide for 10 min. Both NLRP3 (polyclonal NLRP3 antibody; Novus Biologicals, Shanghai) and VEGF (polyclonal VEGF antibody; Abcam, UK) antibodies were used at 1:200 dilution; Collagen I antibody was used at 1:100 dilution (polyclonal Collagen I antibody; Novus Biologicals, Shanghai). Negative controls were performed by omission of the primary antibody. The procedures were performed according to the recommendations of the suppliers, and immunohistochemical staining techniques were performed simultaneously on all samples to reduce inter-assay variability. The expression of NLRP3, VEGF and Collagen I was analyzed semiquantitatively using a visual scoring method in six different parts of lung tissue [9]: alveolar epithelium, bronchiolar epithelium, bronchiolar smoothmuscle, vascular endothelium, vascular smooth muscle and macrophage. The staining intensity was graded and scored from 0 to 3 (0 = no staining; 1 = moderate staining; 2 = intense staining; 3 = extreme intense staining). The mean staining intensity score of each part was recorded by examining four randomly selected areas per each part of lung tissue in one section. Six sections of each time points were randomly selected and scored. The staining score of each sample was recorded by the summation of values of 6 different parts of lung tissue. The scoring was performed by two observers blinded to the experimental data of the subjects. Inter-observer agreement was assessed using weighted kappa statistics and the inter-observer agreement between the two observers for grading staining intensity of lung tissue was excellent (weighted *κ* values > 0.75).

### RNA extraction and reverse transcription

Total RNA was extracted from frozen lung tissues by using miRNeasy Mini Kit (QIAGEN, Germany) following the instructions of the suppliers. The ratio of the readings at 260 nm and 280 nm (A260/A280) was used to determine the purity of RNA and 28S/18S rRNAs were measured to check the integrity of RNA (miRNeasy Mini Handbook, QIAGEN). RNA-based reverse transcription reactions were performed using a High-Capacity cDNA Reverse Transcription Kit with RNase Inhibitor (ABI, US) according to the manufacturer’s instructions.

### Relative quantification of mRNA expression

Rat-specific PCR primers and hydrolysis probes for interleukin-1β (IL-1β), interleukin-6 (IL-6), tumor necrosis factor alpha (TNF-α), surfactant protein A (SP-A), surfactant protein B (SP-B), surfactant protein C (SP-C), NLRP3,VEGF, Collagen I and GAPDH (Life Technologies) were used to perform quantitative PCR reaction on RNA from lung tissue. An additional text file shows this in more detail (see Additional file 1). Ct values of the target genes were normalised to the housekeeping gene GAPDH and expressed as fold changes (ΔCt) relative to pooled control values at each observational time point.

### Western blot analysis for SP-A, SP-B, SP-C, NLRP3, VEGF and collagen I

40 mg lung tissues were lysed directly in 0.5 mL SDS loading buffer, then separated by SDS-PAGE and blotted according to standard protocol. Membranes were probed for SP-A (rabbit anti-rat SP-A;USCN), SP-B (rabbit anti-rat SP- B;USCN), SP-C (rabbit anti-rat SP- C;USCN), NLRP3 (rabbit anti-rat NLRP3;NOVUS), VEGF (rabbit anti-rat VEGF;Abcam), Collagen I (rabbit anti-rat Collagen I;NOVUS), and β-actin as a loading control.

### MicroRNA microarray analysis

Total RNA extracted from frozen lung tissues were prepared and checked as described above. Starting with 1 μg of total RNA, the process began with a Poly (A) Tailing reaction followed by ligation of the biotinylated signal molecule to the target RNA sample (FlashTag Biotin HSR RNA Labeling Kit, Affymetrix, US) according to the manufacturer’s instructions. After preparation of ovens, arrays, and sample registration files, samples were analyzed by the GeneChip miRNA Array (Affymetrix, US) which contains 1218 miRNA probes. GeneChip miRNA array procedure began with hybridization followed by washing, staining, scanning and analysis according to the instructions of Affymetrix GeneChip miRNA Array Procedure. Data analysis was performed by using GeneChip miRNA 2.0 Array 1.1.1.0 software as follows: raw intensity data were imported by setting up robust multi-array analysis (RMA) background detection, RMA correction, quantile normalization, and median polish summarization; analysis of variance (ANOVA) was performed to generate a comprehensive list of differential expressed miRNAs setting a significative *p*-value < 0.01 and a fold change cut-off of 2; Clustering and heatmap analyses were performed by using a software of HemI (Heatmap Illustrator, version 1.0) as it is an excellent method for visualizing multi-dimensional and numerical gene or protein expression data in a single heatmap providing a concise but comprehensive presentation of molecular dynamics under different conditions [10].

The Enzyme Linked Oligosorbent Assay (ELOSA) was designed to provide confirmation that the biotin labeling process and the RNA Spike Control Oligos reaction was working properly. qRT-PCR was also used to verify the results of miRNAs microarray assays.

### Bioinformatic analyses

Target genes of the miRNAs that had significant differential expression between two groups were predicted through an online software of TargetScan (http://www.targetscan.org/vert_71/).

### Statistical analyses

All values were expressed as mean ± standard deviation (SD). *T*-Test was used for measuring the statistical significance of difference between the means of two groups. The chi-squared test was used to determine whether there was a significant difference between frequencies in two groups. ANOVA was performed to generate a comprehensive list of differential expressed miRNAs setting a significative *p*-value < 0.01 and a fold change cut-off of 2. All analyses were performed using SPSS Statistics for Windows (software version 13.0).

## Results

### Intrauterine infection/inflammation rats characteristics

**Fig. 2:**
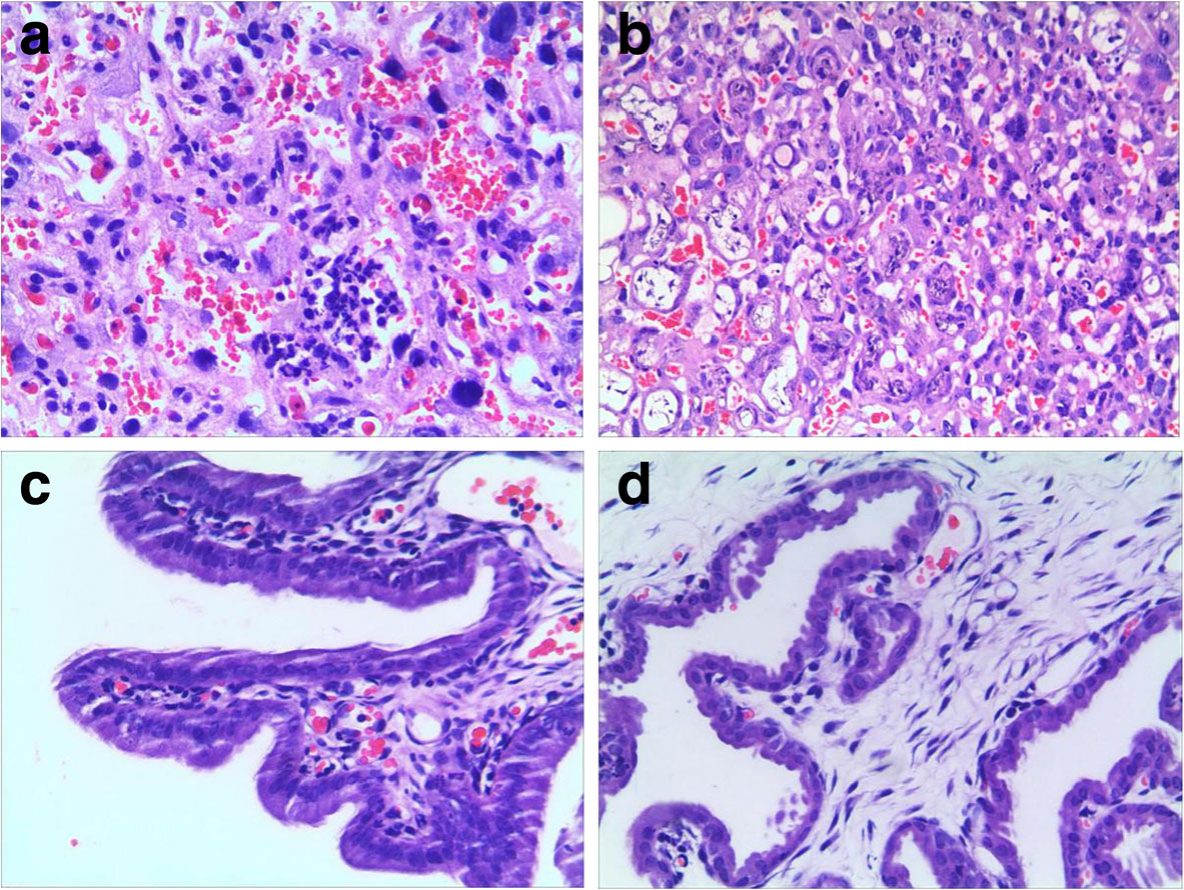
HE Staining for the placenta and uterine wall in SD rats with pregnancies. HE Staining showed vascular congestion, edema, and infiltration of the placenta and uterine wall in intrauterine infection group at E19 (× 100). Panel **a**: the placenta in intrauterine infection group; Panel **b**: Control. Panel **c**: the uterine wall in intrauterine infection group; Panel **d**: Control

There were 24 pregnant SD rats endocervically inoculated with E.coli suspension or sterile normal saline at E15, which had no significant changes in food intake or activity after inoculation. The gestation period of the pregnant SD rats was 21~ 22 days in this study. There were 77 (77/79) (live rat pups/total rat pups) fetal rats and 48 (48/51) neonatal rats in the intrauterine infection group, and 76 (76/78) fetal rats and 52 (52/53) neonatal rats in the control group. There was no significant difference in the mortalities of rat pups between two groups (*χ*^*2*^ = 0.53, *P* > 0.05). Vascular congestion, edema, and marked neutrophil infiltration were manifested in the placenta and uterine wall in intrauterine infection group (Fig. 2). By contrast, there was no manifestation of inflammation in the placenta or uterus in the control group.

**Fig. 3:**
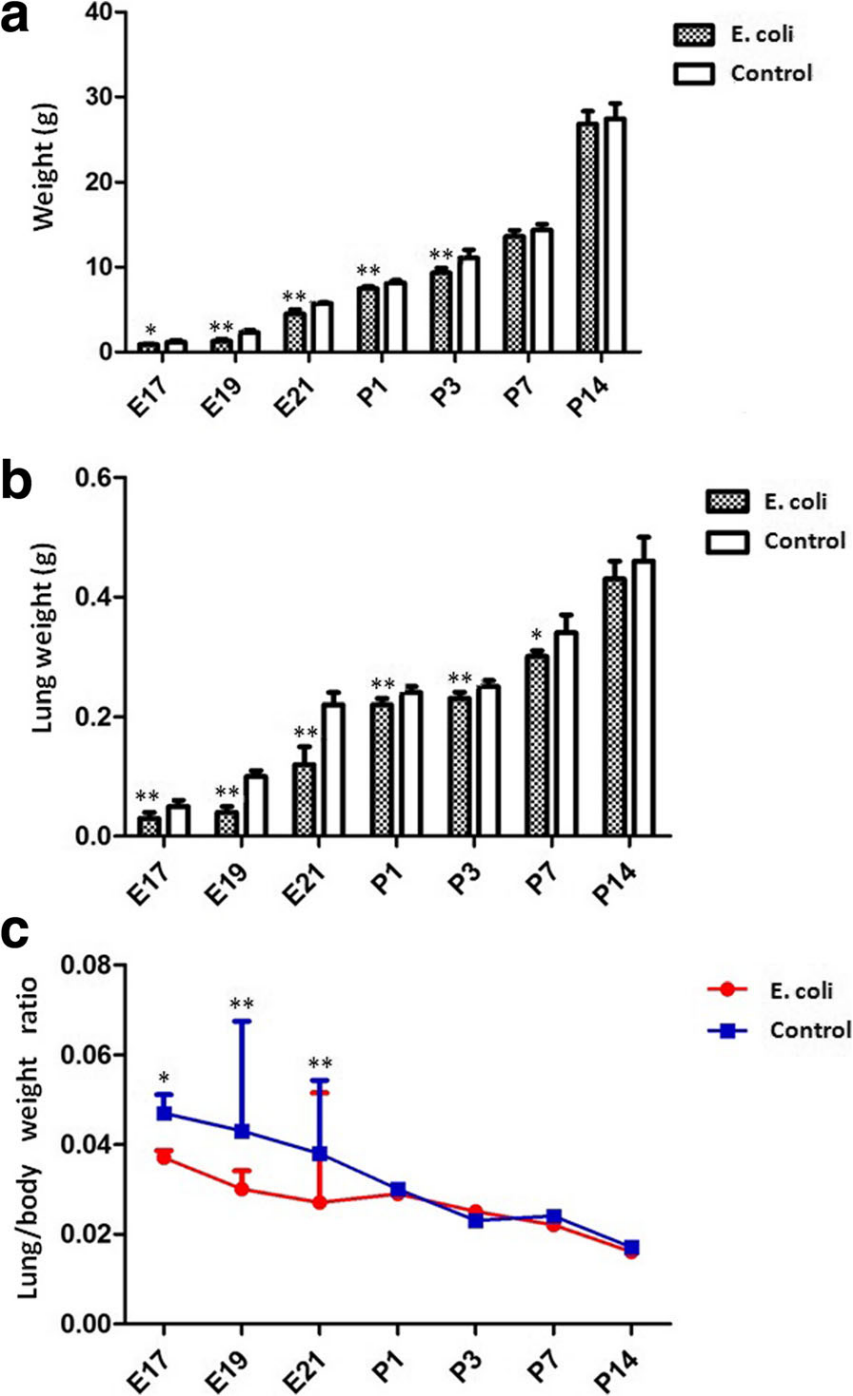
The mean body weights, lung weights and lung/body weight ratios of the fetal and neonatal rats. **a**: The mean body weights of the pups in intrauterine infection group were significantly lower compared to control from E17 to P3. **b**: The mean lung weights of the pups in intrauterine infection group were significantly lower compared to control from E17 to P7. **c**: The mean lung/body weight ratios at E17, E19, E21 in intrauterine infection group were significantly lower compared to the control group. *P < 0.05; **P < 0.01, vs. control group

The mean body weights of the fetal rats at E17, E19, E21 and the neonatal rats at P1, P3 in intrauterine infection group were significantly lower compared to the control group (Fig. 3a). The mean lung weights of the fetal rats at E17, E19, E21 and the neonatal rats at P1, P3, P7 in intrauterine infection group were significantly lower compared to the control group (Fig. 3B). The mean lung/body weight ratios at E17, E19, E21 in intrauterine infection group were significantly lower compared to the control group (Fig. 3c).

### Histomorphological examination of the fetal and neonatal rat lung

**Fig. 4:**
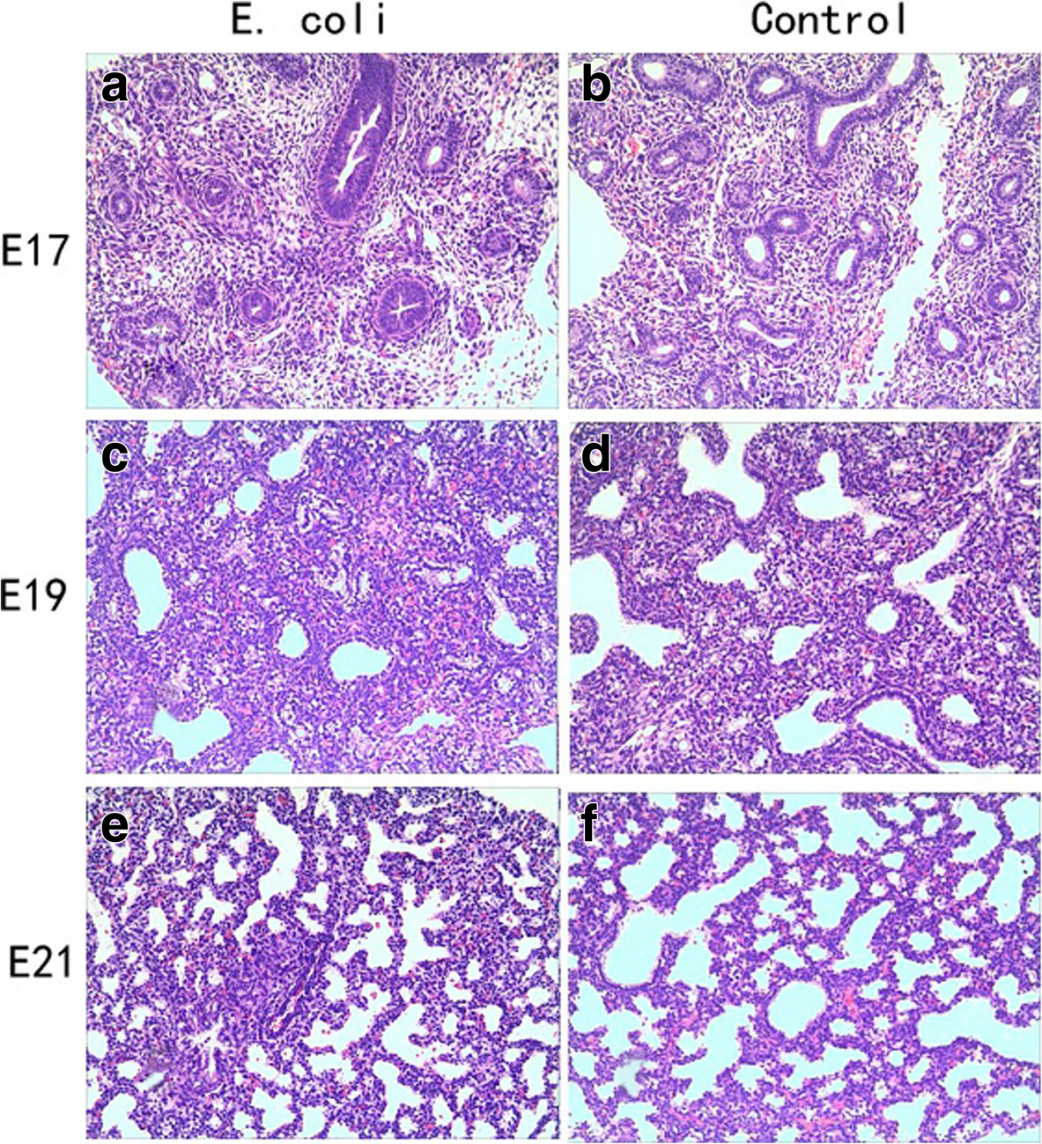
HE Staining for the lungs of fetal rats. Infiltration, less alveolar numbers and thickened alveolar septa of the fetal lung in response to pregnant rat *E. coli* exposure (× 100). **a**: intrauterine infection group at E17; **b**: Control at E17; **c**: intrauterine infection group at E19; **d**: Control at E19; **e**: intrauterine infection group at E21; **f**: Control at E21

**Fig. 5:**
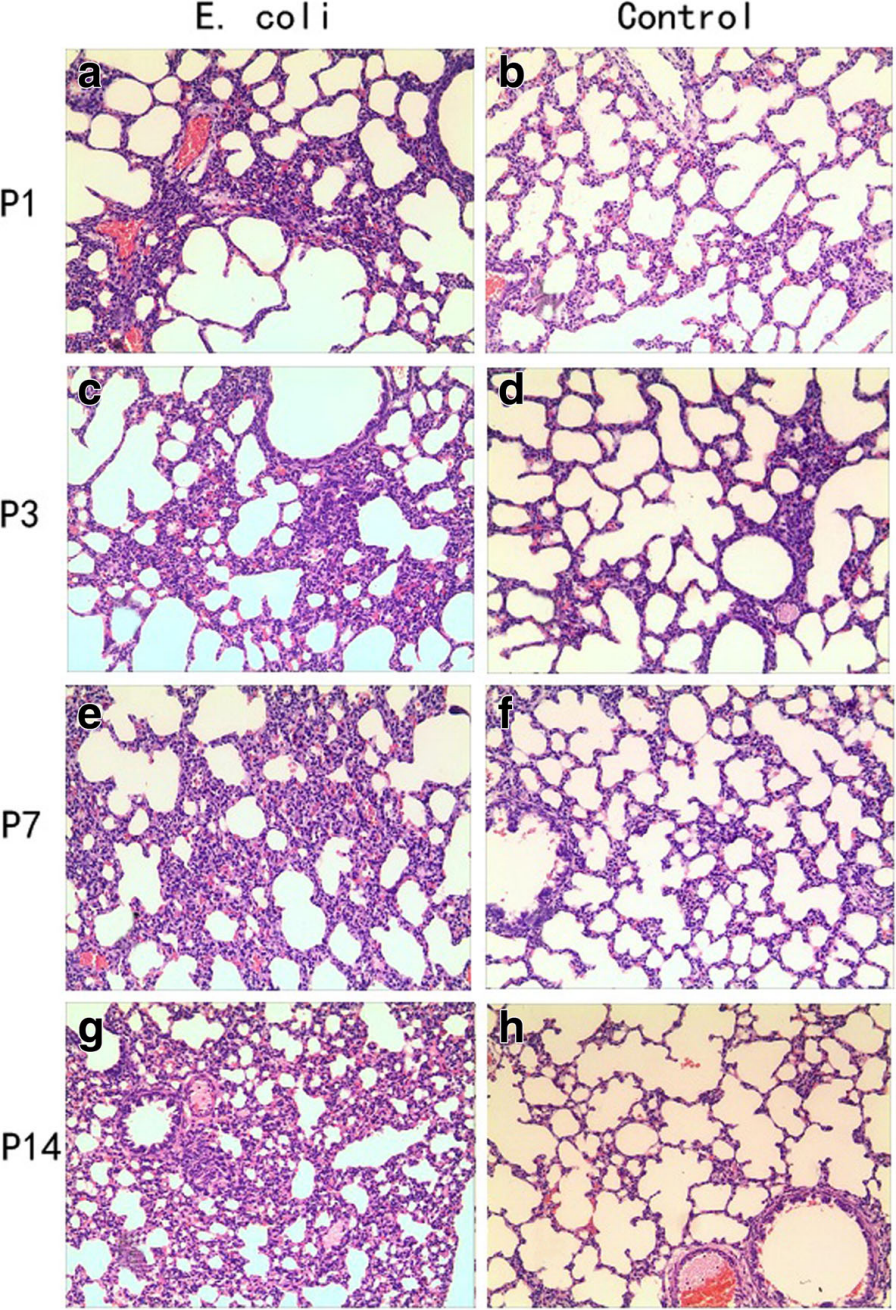
HE Staining for the lungs of neonatal rats. Infiltration, reduced alveolar vesicular structure, less alveolar numbers and thickened alveolar septa of the neonatal lung in response to pregnant rat *E. coli* exposure (× 100). **a**: intrauterine infection group at P1; **b**: Control at P1; **c**: intrauterine infection group at P3; **d**: Control at P3; **e**: intrauterine infection group at P7; **f**: Control at P7; **g**: intrauterine infection group at P14; **h**: Control at P14

The fetal and neonatal rat lung tissues histomorphological examination showed inflammatory infiltration, reduced alveolar vesicular structure, less alveolar numbers and thickened alveolar septa in intrauterine infection group compared to the control group. Representative examples of histomorphological examination of the fetal and neonatal rat lung tissues are shown in Figs. 4 and 5.

### Immunohistochemical analysis for NLRP3, VEGF, and collagen I

**Fig. 6:**
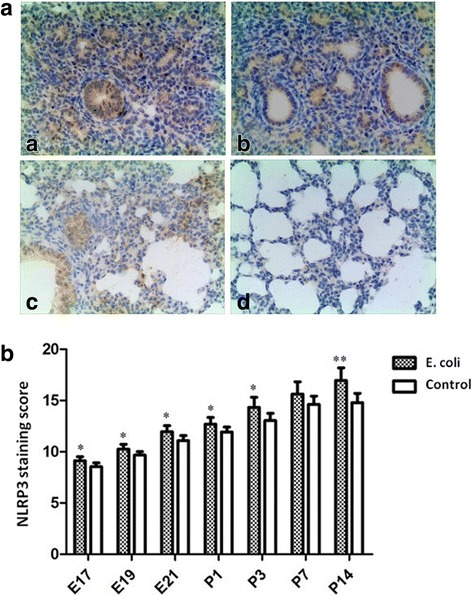
Immunohistochemical staining for NLRP3. A Representative photographs of immunohistochemical staining for NLRP3 in the fetal and neonatal rat lung. The brownish areas indicate where NLRP3 is expressed (× 200). **a**: intrauterine infection group at E19; **b**: Control at E19; **c**: intrauterine infection group at P14; **d**: Control at P14. B Staining scores of NLRP3 in the lung tissue. Comparing intrauterine infection group with control, NLRP3 expression of lung tissue was significantly increased in the fetal rats at E17, E19, E21 and the neonatal rats at P1, P3, P14. *P < 0.05; **P < 0.01, vs. control group

**Fig. 7:**
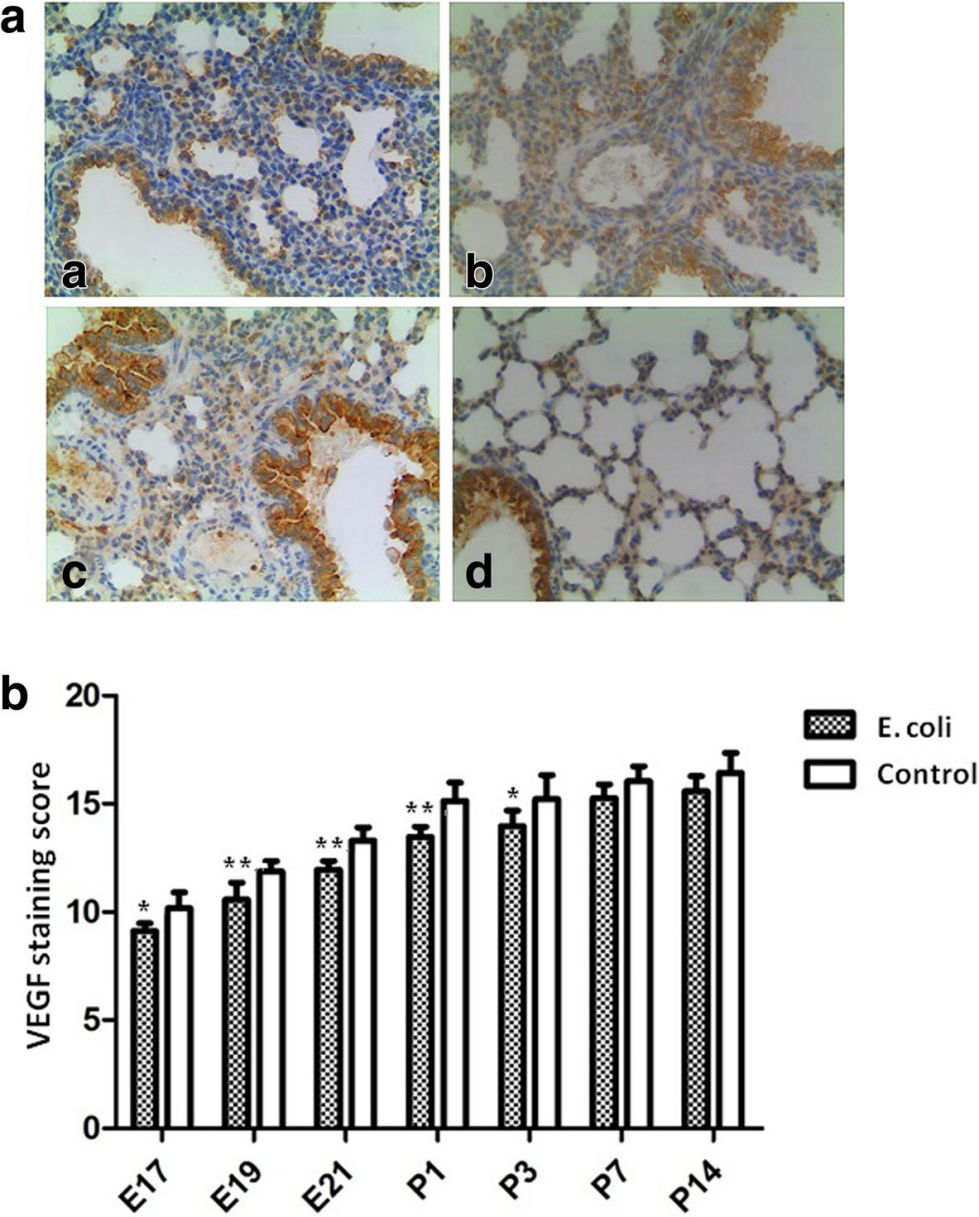
Immunohistochemical staining for VEGF. A Representative photographs of immunohistochemical staining for VEGF in the fetal and neonatal rat lung. The brownish areas indicate where VEGF is expressed (× 200). **a**: intrauterine infection group at E21; **b**: Control at E21; **c**: intrauterine infection group at P14; **d**: Control at P14. B Staining scores of VEGF in the lung tissue. Comparing intrauterine infection group with control, VEGF expression of lung tissue was significantly decreased in the fetal rats from E17 to P3. *P < 0.05; **P < 0.01, vs. control group

Examples of immunohistochemical staining for NLRP3 are presented in Fig. 6a. According to the semi-quantitative scoring method, NLRP3 expression of lung tissue was significantly increased in the fetal rats at E17, E19, E21 and the neonatal rats at P1, P3, P14 in intrauterine infection group compared to the control group (Fig. 6b). Figure 7a shows a representative example of immunohistochemical staining for VEGF in the fetal and neonatal rat lung tissue. According to the semi-quantitative scoring method, VEGF expression of lung tissue was significantly decreased in the fetal rats at E17, E19, E21 and the neonatal rats at P1, P3 in intrauterine infection group compared to the control group (Fig. 7b).

**Fig. 8:**
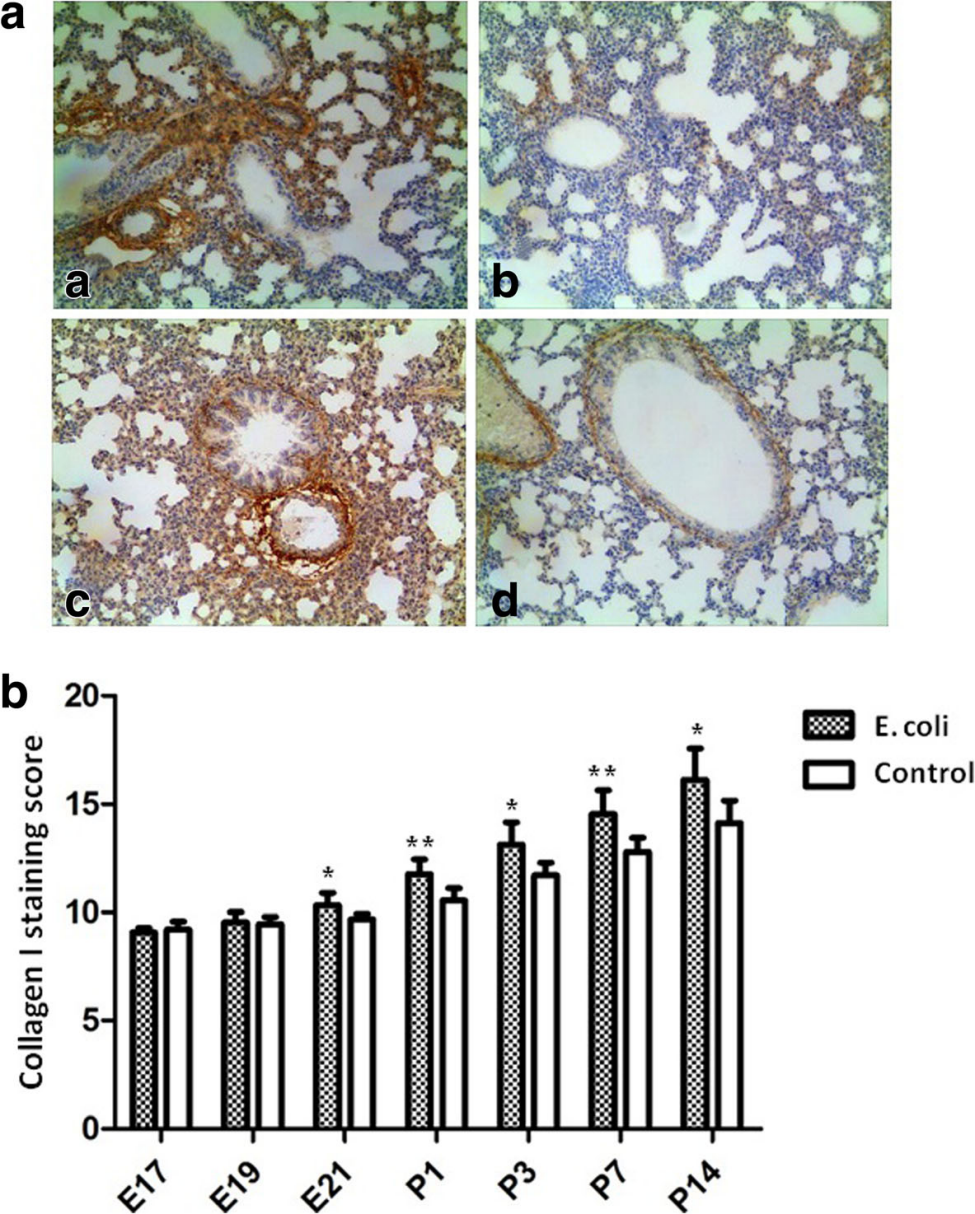
Immunohistochemical staining for Collagen I. A Representative photographs of immunohistochemical staining for Collagen I in the fetal and neonatal rat lung. The brownish areas indicate where Collagen I is expressed (× 200). **a**: intrauterine infection group at E21; **b**: Control at E21; **c**: intrauterine infection group at P14; **d**: Control at P14. B Staining scores of Collagen I in the lung tissue. Comparing intrauterine infection group with control, Collagen I expression of lung tissue was significantly increased from E21 to P14. *P < 0.05; **P < 0.01, vs. control group

Representative examples of immunohistochemical staining for Collagen I are shown in Fig. 8a. According to the semi-quantitative scoring method, Collagen I expression of lung tissue was significantly increased in the fetal rats at E21 and the neonatal rats at P1, P3, P7, P14 in intrauterine infection group compared to the control group (Fig. 8b).

### mRNA expression of IL-1β, IL-6, TNF-α, NLRP3, SP-A, SP-B, SP-C, VEGF and collagen I genes

**Fig. 9:**
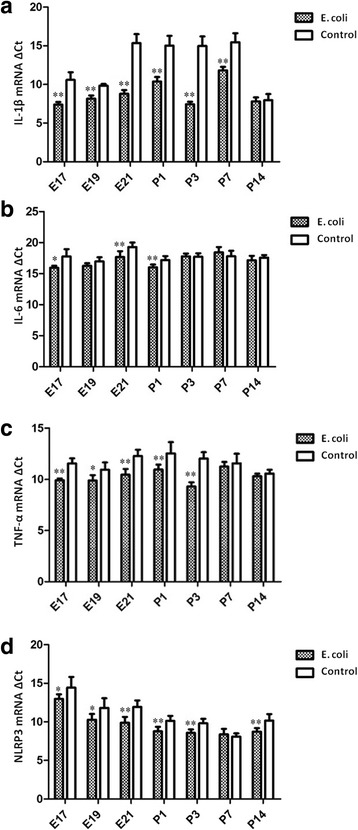
mRNA expression of IL-1β, IL-6, TNF-α and NLRP3. **a**: Analysis of IL-1β expression in the fetal and neonatal rat lung tissues showed significant mRNA up-regulation at E17, E19, E21 d and P1, P3, P7 in intrauterine infection group, relative to control. **b**: qRT-PCR analysis for the fetal and neonatal rats lung tissues showed significantly increased expression of IL-6 at E17, E21and P1 in intrauterine infection group, relative to control. **c**: Analysis of TNF-α expression in the fetal and neonatal rat lung tissues showed significant mRNA up-regulation at E17, E19, E21 and P1, P3 in intrauterine infection group, relative to control. **d**: Significant increases in NLRP3 (at E17, E19, E21 and P1, P3, P14) mRNA expression were identified in intrauterine infection group compared to the control group. The mRNA quantitation was calculated by using the ΔCt method and higher ΔCt means lower expression levels. *P < 0.05; **P < 0.01, vs. control group

Analysis of cytokine (IL-1β, IL-6, TNF-α and NLRP3) expression in the fetal and neonatal rat lung tissue identified a pattern of significant mRNA up-regulation in intrauterine infection group at different time points, relative to control. Significant increases in IL-1β (at E17, E19, E21 d and P1, P3, P7), IL-6 (at E17, E21and P1), TNF-α (at E17, E19, E21 and P1, P3) and NLRP3 (at E17, E19, E21 and P1, P3, P14) mRNA expression were identified in intrauterine infection group compared to the control group (Fig. 9).

**Fig. 10:**
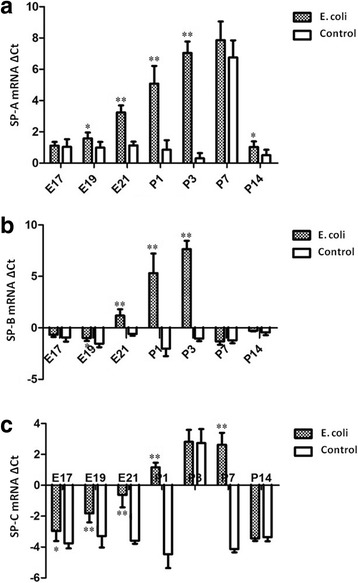
mRNA expression of SP-A, SP-B and SP-C. **a**: Analysis of SP-A expression in the fetal and neonatal rat lung tissues showed significant mRNA down-regulation at E19, E21and P1, P3, P14 in intrauterine infection group, relative to control. **b**: Significant decreases in SP-B (at E19, E21 and P1, P3) mRNA expression were identified in intrauterine infection group compared to the control group. **c**: qRT-PCR analysis for the fetal and neonatal rats lung tissues showed significantly decreased expression of SP-C at E17, E19, E21and P1, P7 in intrauterine infection group, relative to control. The mRNA quantitation was calculated by using the ΔCt method and higher ΔCt means lower expression levels. *P < 0.05; **P < 0.01, vs. control group

Analysis of pulmonary surfactant proteins (SP-A, SP-B and SP-C) expression in the fetal and neonatal rat lung tissue identified a pattern of significant mRNA down-regulation in intrauterine infection group at different time points, relative to control. Significant decreases in SP-A (at E19, E21and P1, P3, P14), SP-B (at E19, E21and P1, P3) and SP-C (at E17, E19, E21and P1, P7) mRNA expression were identified in intrauterine infection group compared to the control group (Fig. 10).

**Fig. 11:**
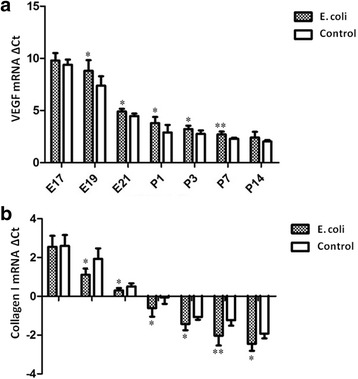
mRNA expression of VEGF and Collagen I. qRT-PCR analysis for the fetal and neonatal rats lung tissues showed significantly decreased expression of VEGF at E19, E21and P1, P3, P7 in intrauterine infection group, relative to control **a**. Analysis of Collagen I expression in the fetal and neonatal rat lung tissues showed significant mRNA up-regulation at E19, E21 and P1, P3, P7, P14 in intrauterine infection group, relative to control **b**. The mRNA quantitation was calculated by using the ΔCt method and higher ΔCt means lower expression levels. *P < 0.05; **P < 0.01, vs. control group

qRT-PCR analysis for the fetal and neonatal rats lung tissues showed significantly decreased expression of VEGF at E19, E21and P1, P3, P7 in intrauterine infection group, relative to control (Fig. 11a). Analysis of Collagen I expression in the fetal and neonatal rat lung tissue showed significant mRNA up-regulation at E19, E21 and P1, P3, P7, P14 days after birth in intrauterine infection group, relative to control (Fig. 11b).

### Western blot analysis for SP-A, SP-B, SP-C, NLRP3, VEGF and collagen I

**Fig. 12:**
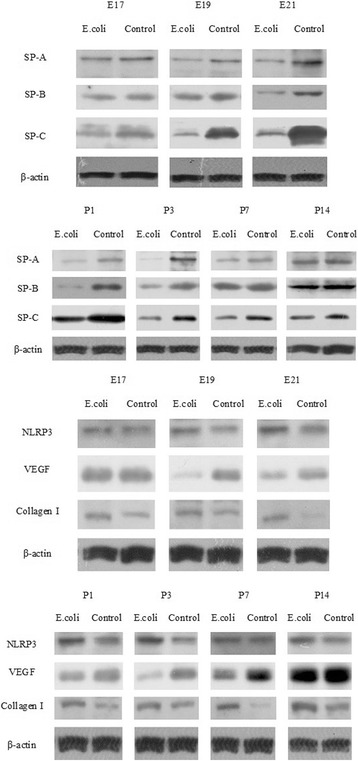
Western blotting for SP-A, SP-B, SP-C, NLRP3, VEGF, Collagen I and β-actin from the fetal and neonatal rat lung. E.coli represents intrauterine infection group

Examples of western blot for SP-A, SP-B, SP-C, NLRP3, VEGF and Collagen I are presented in Fig. 12.

**Fig. 13:**
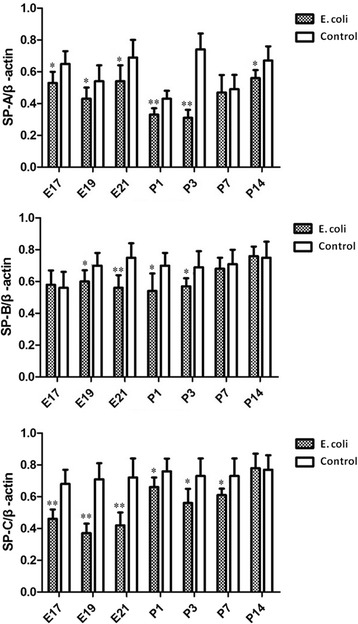
Quantification of SP-A, SP-B and SP-C from the fetal and neonatal rat lung. Densitometric analysis revealed that pulmonary surfactant proteins including SP-A (at E17, E19, E21and P1, P3, P14), SP-B (at E19, E21and P1, P3) and SP-C (at E17, E19, E21and P1, P3, P7) were significantly decreased in intrauterine infection group compared to the control group. *P < 0.05; ** *P* < 0.01, vs. control group.

Densitometric analysis revealed that pulmonary surfactant proteins including SP-A (at E17, E19, E21and P1, P3, P14), SP-B (at E19, E21and P1, P3) and SP-C (at E17, E19, E21and P1, P3, P7) were significantly decreased in intrauterine infection group compared to the control group (Fig. 13).

**Fig. 14:**
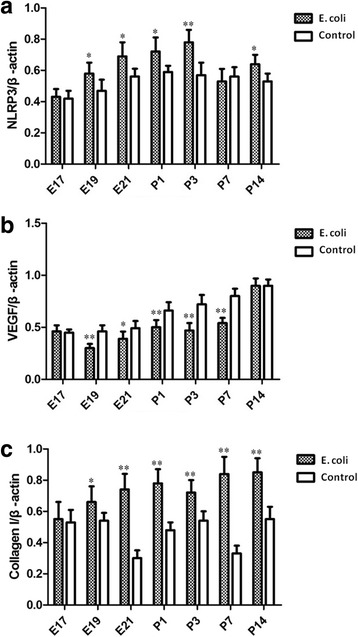
Quantification of NLRP3, VEGF and Collagen I from the fetal and neonatal rat lung. NLRP3 expression was significantly increased at E19, E21 and P1, P3, P14 in intrauterine infection group compared with control while it was not significantly different between the two groups at E17, P7 **a**. Western blot assays of the fetal and neonatal rats lung tissues showed VEGF expression was significantly decreased at E19, E21 and P1, P3, P7 in intrauterine infection group compared with control while it was not significantly different between the two groups at E17 and P14 **b**. Densitometric analysis showed Collagen I expression was significantly increased in intrauterine infection group compared with control, except at E17 **c**. *P < 0.05; **P < 0.01, vs. control group

NLRP3 expression was significantly increased at E19, E21 and P1, P3, P14 in intrauterine infection group compared with control while it was not significantly different between the two groups at E17, P7 (Fig. 14a). Western blot assays of the fetal and neonatal rats lung tissues showed VEGF expression was significantly decreased at E19, E21 and P1, P3, P7 in intrauterine infection group compared with control while it was not significantly different between the two groups at E17 and P14 (Fig. 14b). Densitometric analysis showed Collagen I expression was significantly increased in intrauterine infection group compared with control, except at E17 (Fig. 14c). The results of Western blot assays were consistent with those of the immunohistochemical staining and qRT-PCR.

### Microarray profile of neonatal rat lung tissues

**Fig. 15:**
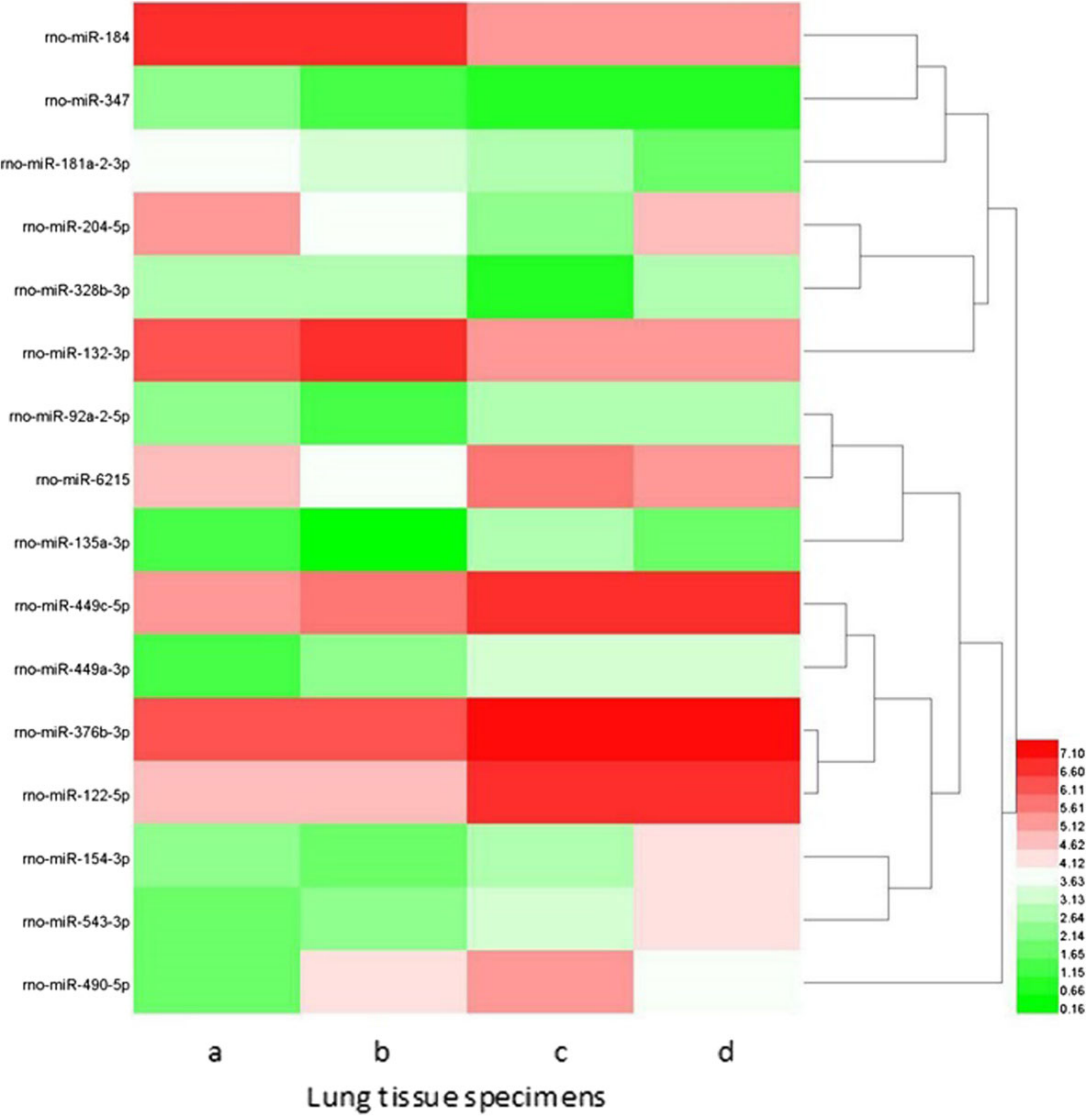
Hierarchical clusterings analysis of miRNA expression profile for intrauterine infection group and control at P1. **a**: Specimen a was collected from intrauterine infection group at P1. **b**: Specimen b was also collected from intrauterine infection group at P1. **c**: Specimen c was collected from the control group at P1. **d**: Specimen d was collected from the control group at P1. Red colour represents an expression level above mean (white colour), and green colour represents expression lower than the mean. rno-miR-184, rno-miR-347, rno-miR-181a-2-3p, rno-miR-204-5p, rno-miR-132-3p and rno-miR-328b-3p are significantly up-regulated while other 10 miRNAs are significantly down-regulated

**Fig. 16:**
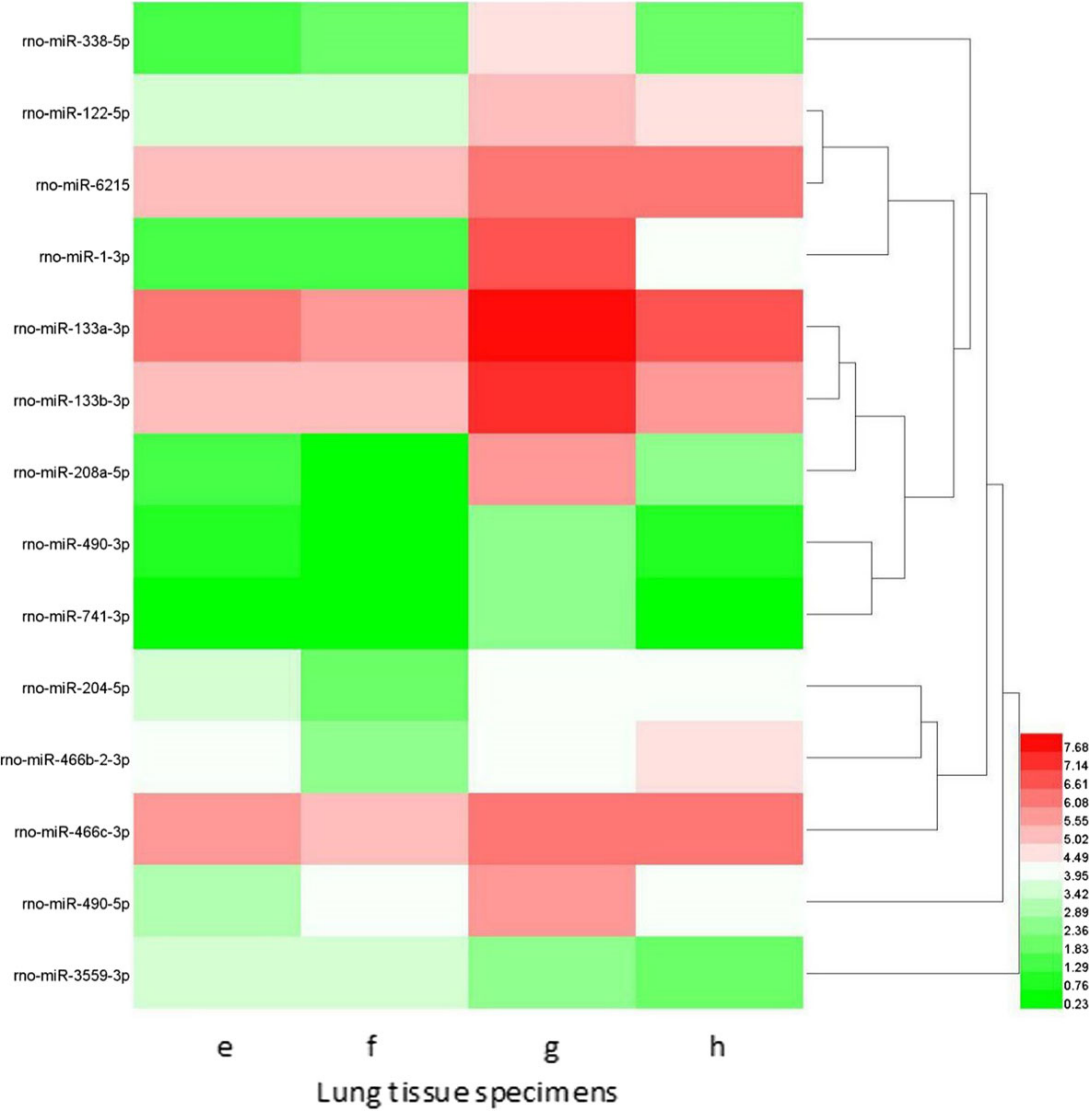
Hierarchical clusterings analysis of miRNA expressionprofile for intrauterine infection group and control at P3. Columns represent lung tissue specimens and rows represent miRNAs. Specimen e and f were collected from intrauterine infection group, and specimen g and h were collected from the control group. Rno-miR-3559-3p is significantly up-regulated while other 13 miRNAs are significantly down-regulated

**Fig. 17:**
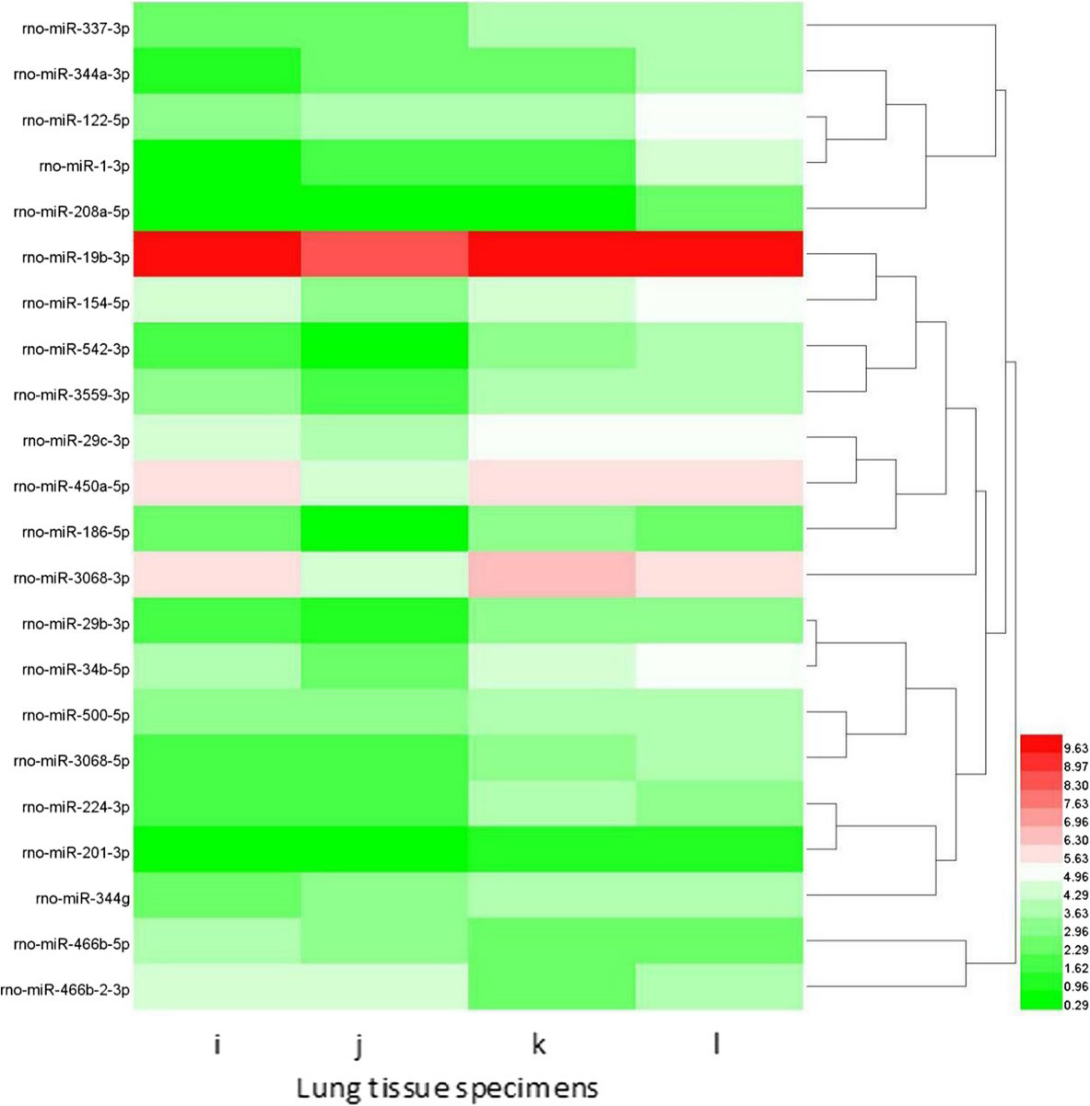
Hierarchical clusterings analysis of miRNA expression profile for intrauterine infection group and control at P14. Columns represent lung tissue specimens and rows represent miRNAs. Specimen i and j were collected from intrauterine infection group, and specimen k and l were collected from the control group. Rno-miR-466b-2-3p and rno-miR-466b-5p are significantly up-regulated while other 20 miRNAs are significantly down-regulated

**Fig. 18:**
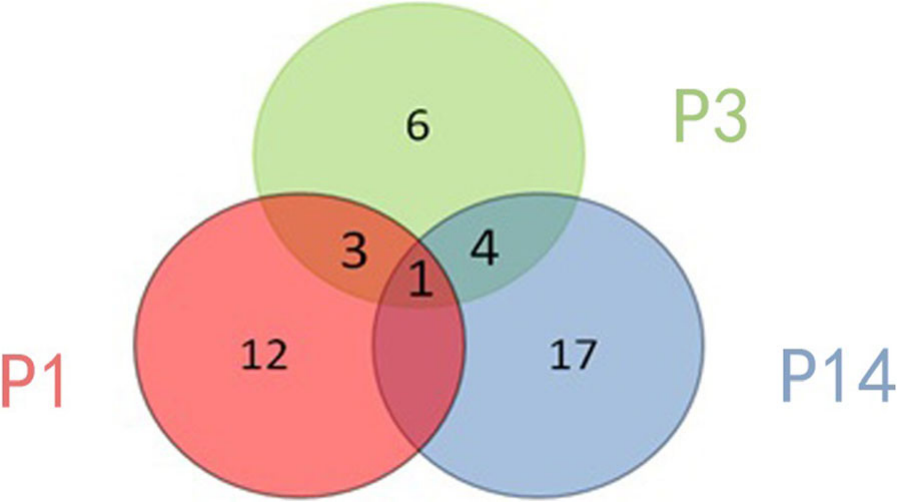
Venn diagrams for identified miRNAs. Venn diagrams summarizing the number of miRNAs found to be differentially expressed by comparing intrauterine infection group with control at P1 (red), P3 (green) and P14 (blue). Number for conjoint (and non-conjoint) differentially expressed miRNAs are also indicated

The 6 neonatal rat lung tissue samples in intrauterine infection group (every 2 samples at P1, P3, P14 respectively) and 6 normal neonatal rat lung tissues (every 2 samples at P1, P3, P14 respectively as control) were profiled on the Affymetrix GeneChip miRNA 2.0 Arrayanalysis demonstrated that there were some miRNA expression differences between the intrauterine infection group and control at P1, P3 and P14. By using ANOVA model, 16 miRNAs (6 significantly upregulated and 10 significantly downregulated) were found differentially expressed in intrauterine infection group vs control at P1, 14 (1 significantly upregulated and 13 significantly downregulated) in intrauterine infection group vs control at P3, and 22 (2 significantly upregulated and 20 significantly downregulated) in intrauterine infection group vs control at P14. Hierarchical clusterings of the miRNAs identified by comparing intrauterine infection group and control at P1, P3, P14 are shown in Figs. 15, 16, and 17 In addition, logical relations resulting from our three-sets Venn Diagram show shared miRNAs which coherently differentiate intrauterine infection group and control (Fig. 18).

### miRNA analytical validation by qRT-PCR

**Fig. 19:**
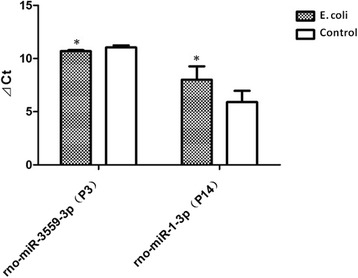
The mRNA quantitation for rno-miR-3559-3p and rno-miR-1-3p. rno-miR-3559-3p was significantly up-regulated at P3 and rno-miR-1-3p was significantly down-regulated at P14 in intrauterine infection group compared to control. The mRNA quantitation was calculated by using the ΔCt method and higher ΔCt means lower expression levels. *P < 0.05, vs. control group

To validate the differential expression of the miRNAs identified by array profiling, the 10 neonatal rat lung tissue samples in intrauterine infection group (every 5 samples at P3, P14 respectively) and 10 normal controls were analyzed by qRT-PCR for rno-miR-3559-3p and rno-miR-1-3p which were selected randomly from the results of miRNA microarray analysis. qRT-PCR analyses of the fetal and neonatal rat lung tissue samples showed that rno-miR-3559-3p was significantly up-regulated at P3 in intrauterine infection group compared to control, and rno-miR-1-3p was significantly down-regulated at P14 in intrauterine infection group compared to control (Fig. 19). Both results were consistent with the results of miRNA microarray analysis.

### Target genes of the identified miRNAs

**Fig. 20:**
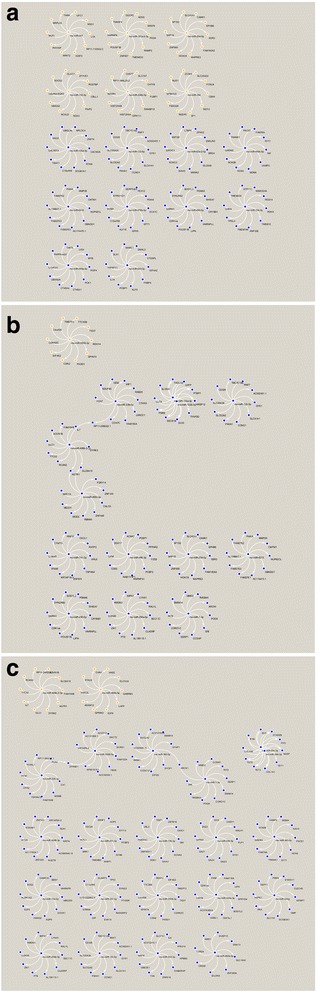
Interaction networks of identified miRNAs and target genes in neonatal rats after intrauterine infection/inflammation. **a**: P1, **b**: P3, **c**: P14. Orange color represents up-regulated miRNAs and their target genes, and blue color represents down-regulated miRNAs and their target genes

Target genes of the 43 identified miRNAs were predicted through the online bioinformatic software of TargetScan. The predicted target genes that probably express the most effectively targeted mRNAs are very rich and providing very useful clues for further study on these miRNAs (Fig. 20).

## Discussion

Intrauterine infection plays an important role in the development of BPD [11, 12]. Comparatively little animal data are available to describe the precise molecular biological mechanisms of intrauterine infection/inflammation on fetal and neonatal lung development. Our research presents novel data of the characteristics of lung development and miRNAs expression in developmental phases of lungs with injury after intrauterine infection/inflammation. These findings add weight to recent clinical researches concluding that the prompt diagnosis and treatment of intrauterine infection/inflammation is critical to lung development [13, 14]. These findings also provide experimental basis for clinical prevention of BPD.

HE Staining showed vascular congestion, edema, and neutrophil infiltration in the placenta and uterine wall in intrauterine infection group while there was no manifestation of inflammation in the placenta or uterus in the control group. This observation suggests an animal model of intrauterine infection/inflammation is successfully established with pregnant SD rats endocervically inoculated with E.coli suspension. To mimic intrauterine infection, lipopolysaccharide (LPS) or inactivated *E. coli* is commonly injected into the uterus, which is invasive, and can cause premature birth in control animals [15, 16]. In order to mimic natural pathophysiological process, *E. coli* was used and less-invasive vaginal dilator-guided endocervically intramuscular injections were performed for the animal model of intrauterine infection/inflammation in this study. The period of antenatal development of human lung have traditionally been divided into five stages: embryonic stage (0–7 weeks in utero), pseudoglandular stage (7–17 weeks in utero), canalicular stage (17–27 weeks in utero), saccular stage (28–36 weeks in utero), alveolar stage (36 weeks in utero-birth) [17]. BPD is mainly seen in premature infants born at the canalicular and saccular stages of lung development. The lung development of the E17, E19, and E21 rat pups is at the canalicular and saccular stages [18, 19]. The fetal and neonatal rat lung tissues histomorphological examination showed inflammatory infiltration, reduced alveolar vesicular structure, less alveolar numbers and thickened alveolar septa in intrauterine infection group compared to the control group. These pathological changes were very similar to those observed in preterm infants with pneumonitis and BPD [20].

The mean body weights of the pup rats were significantly lower in intrauterine infection group compared to the control group from E17 to P3. These data suggest intrauterine infection/inflammation has a negative effect on the growth and development of fetal and neonatal rats which is similar to intrauterine growth retardation (IUGR). The mechanism may be that intrauterine infection leads to chorioamnionitis and fetal inflammatory response syndrome (FIRS), which affects the absorption and utilization of fetal nutrition. In the intrauterine infection group, the lung weight was significantly lower than that of the control group from E17 to P7. The mean lung/body weight ratios in intrauterine infection group were significantly lower compared to the control group at E17, E19, E21, but there was no significant difference between the two groups after birth. These results show intrauterine infection/inflammation impairs the lung development of fetal and neonatal rats, especially the lung development of fetal rats.

Previous research has suggested that VEGF and its receptors may be involved in lung inflammatory damage, resulting in acute and chronic lung injury in preterm infants (such as RDS, BPD, etc.) [21–23]. The intensity of immunohistochemical staining showed VEGF expression of lung tissue was significantly decreased in the fetal rats and the neonatal rats at P1, P3 in intrauterine infection group compared to the control group. The results of RT-PCR and Western blot also showed that the expression of VEGF in lung tissue was decreased after intrauterine infection/inflammation. Although the results are generally consistent with previous research, these findings suggest VEGF expression is decreased in the fetal and neonatal lung tissue after intrauterine infection/inflammation initiated at the pseudoglandular stage.

Collagen I is an important extracellular matrix component in lung tissue. Its synthesis will be enhanced during lung injury and remodeling (such as infection, inflammation and fibrosis) [24–26]. The results of immunohistochemical staining, RT-PCR and Western blot showed that the expression of CollagenI in the fetal and neonatal lung in the intrauterine infection group was significantly higher than that of the control group at several observation time points. These results reveal that injury and remodeling of lung tissue after intrauterine infection/inflammation are obvious, which results in abnormal lung development in fetal and neonatal rats.

NLRP3 is the best studied NOD-like receptor (NLR) thus far, activated in response to both exogenous and endogenous molecules. Recently, the NLRP3 inflammasome was implicated in a mouse model of hyperoxia-induced BPD [4]. Our results of immunohistochemical staining, RT-PCR and Western blot show that the expression of NLRP3 inflammasome is increased in the fetal and neonatal lung injury after intrauterine infection/inflammation and the levels of inflammatory factors IL-1β and IL-6 were up regulated. These data reveal the NLRP3 inflammasome is critically involved in the development of BPD.

Surfactant proteins are important indicators of lung maturity. Previous study showed that intrauterine infection/inflammation can promote the expression of pulmonary surfactant proteins, promoting lung maturation [27, 28]. But our results of RT-PCR and Western blot show that the expression of pulmonary surfactant proteins including SP-A, SP-Band SP-C is decreased in the fetal and neonatal lung injury after intrauterine infection/inflammation. It may be related to the different kinds of experimental animals and the different severity of intrauterine infection/inflammation.

miRNAs are endogenous non-coding single stranded RNAs with about 22 nucleotides in length that post-transcriptionally regulate gene expression. To date, some studies have investigated miRNAs expression in lung injury, but they mainly focused on limited kinds of miRNAs [29–31]. In this study we compared the miRNA expression profile of 6 lung specimens collected from intrauterine infection group with those collected from the control group at different time points. We found 43 miRNAs differentially expressed in the two groups; the results of validation by RT-PCR were consistent with those of miRNA microarray analysis. Number for conjoint (and non-conjoint) differentially expressed miRNAs are also indicated, and some miRNAs play an important role at different time points.

Our data showing that differentially expressed miRNAs can target a wide range of genes suggests that miRNAs may play an important role in the development of lung injury after intrauterine infection/inflammation. Although this remains to be further studied, differentially expressed 43 miRNAs may be associated with the development of BPD. This is true in the development of BPD, where some miRNAs have been identified [32–34], but the molecular mechanism by which the miRNAs post-transcriptionally regulate target gene expression is not yet clear.

From a preventive perspective, it might be interesting to monitor some of the identified miRNAs as novel biomarkers if the miRNAs are also suitable to be used to predict individual BPD risk. This would be more appropriate if an miRNA/target gene imbalance is identified in BPD. In addition, the identified miRNAs could themselves become targets with antisense therapeutics to recover intrauterine infection/inflammation or reduce the occurrence of lung injury and BPD.

The results of our study showed significant changes of pulmonary cytokine expression and morphology in both fetal rats and neonatal rats in intrauterine infection group compared to the control group at different observation time point. These results suggest that effects of intrauterine infection/inflammation including pulmonary inflammatory response and remodeling may persist beyond the fetal period and continue into the neonatal period. The reason why these effects of intrauterine infection/inflammation persist for such a long time may be related to alterations in the activities of the NLRP3 inflammasome and miRNAs driving an excessive inflammatory response, and it needs to be further studied. Exposure to oxygen and/or ventilation, particularly in preterm infants exposed to chorioamnionitis or intrauterine infection/inflammation, results in an exacerbation of pulmonary inflammatory influx and remodeling with subsequent development of BPD.

Our study has a few limitations. First, the animal model which was established with SD rats is a small animal model. It could limit the study of the multi-hit theory for BPD as mechanical ventilation, hyperoxic toxicity, invasive procedure and parenteral nutrition are unfeasible in a small animal model. Second, the study has not included molecular function study because it is difficult to perform any surfactant bio-physical study or bronchoalveolar lavage sampling in these small animals. Third, the study has not explored the effect of intrauterine infection/inflammation on the lung development in the very early fetal stage.

## Conclusions

Our research yielded three major conclusions. First, intrauterine infection/inflammation not only results in significantly decreased body weight, lung weight and lung/body weight ratio in the fetal and neonatal rats but also leads to reduced alveolar vesicular structure, less alveolar numbers and thickened alveolar septa, which are very similar to the changes observed in the new BPD. Second, possible mechanisms of intrauterine infection/inflammation on fetal and neonatal lung development are very complex,which may include NLRP3 inflammasome activation followed by inflammatory cytokines expression up-regulated, inflammatory damage, inhibiting the expression of pulmonary surfactant proteins, interfering with lung interstitial development. Third, there were 43 miRNAs identified by miRNAs microarray assays, which target a wide range of genes and may play an important role in regulating the processes of BPD after intrauterine infection/inflammation.

## Additional file

Additional file 1: Relative quantification of mRNA expression. (DOCX 15 kb)

## Abbreviations

BPD: bronchopulmonary dysplasia
FIRS: fetal inflammatory response syndrome
IUGR: intrauterine growth retardation
NLRP3: NOD-like receptor family, pyrin domain containing 3
SP: surfactant protein
VEGF: vascular endothelial growth factor
