# Supplementary material for: Effects and molecular mechanisms of intrauterine infection/inflammation on lung development
Source: Respir Res. 2018 May 10;19:93. doi: 10.1186/s12931-018-0787-y (PMC5946538; doi:10.1186/s12931-018-0787-y)
Supplement: Supplementary file 1 — Relative quantification of mRNA expression. (DOCX 15 kb) [file 12931_2018_787_MOESM1_ESM.docx]

**Additional file 1. Relative quantification of mRNA expression**

Rat-specific PCR primers (table 1) and hydrolysis probes for interleukin-1β (IL-1β), interleukin-6 (IL-6), tumor necrosis factor alpha (TNF-α), surfactant protein A (SP-A), surfactant protein B (SP-B), surfactant protein C (SP-C), NLRP3, VEGF, Collagen I and GAPDH (Life Technologies) were used to perform quantitative PCR reactions on RNA from lung tissue. Reactions were performed by real-time quantitative fluorescent PCR (ABI PRISM7500 Sequence Detection System, Applied Biosystems, US) with 10 ng template RNA in a total volume of 20 μL. Reaction cycling conditions for IL-1β, IL-6, TNF-α, SP-A, SP-B and SP-C were as follows: thermal mixing and hot start phase at 50°C for 2 min and at 95°C for 2 min, followed by 40 cycles of 95°C for 15 s and 60°C for 30 s. Reaction cycling conditions for NLRP3, VEGF and Collagen I were as follows: an initial denaturation at 95°C for 10 min, followed by 40 cycles of 95°C for 15 s and 60°C for 1 min.

**Table 1 Rat-specific PCR primers sequences for IL-1β, IL-6, TNF-α, SP-A, SP-B, SP-C, NLRP3, VEGF, Collagen I and GAPDH**

| **Main target** |  | **Sequence (5′ → 3′)** | **PubMed gene ID** |
| --- | --- | --- | --- |
| IL-1β | Forward | CTATGGCAACTGTCCCTGAA | 24494 |
|  | Reverse | GGCTTGGAAGCAATCCTTAATC |  |
| IL-6 | Forward | CTTCACAAGTCGGAGGCTTAAT | 24498 |
|  | Reverse | GCATCATCGCTGTTCATACAATC |  |
| TNF-α | Forward | ACCTTATCTACTCCCAGGTTCT | 24835 |
|  | Reverse | GGCTGACTTTCTCCTGGTATG |  |
| SP-A | Forward | GCCTTGACCATGAGATGGATAG | 24773 |
|  | Reverse | GAGGGCAGCACTTAGTTTAGAG |  |
| SP-B | Forward | GTGCCAAGAGTGTGAGGATATT | 192155 |
|  | Reverse | ACAAGCAGCTTCAAGGGTAG |  |
| SP-C | Forward | GGGTAGCAAAGAGGTACTGATG | 50683 |
|  | Reverse | ACCACAACCACGATGAGAAG |  |
| NLRP3 | Forward | CTTCAGGCTGATCCAAGAGAAT | 287362 |
|  | Reverse | CAGTCTCCATCTGCTGCTTTA |  |
| VEGF | Forward | GTGAATGCAGACCAAAGAAAGATAG | 83785 |
|  | Reverse | CAGTGAACGCTCCAGGATTTA |  |
| Collagen I | Forward | CAAGATGGTGGCCGTTACTAC | 29393 |
|  | Reverse | GCTGCGGATGTTCTCAATCT |  |
| GAPDH | Forward | ACTCCCATTCTTCCACCTTTG | 24383 |
|  | Reverse | CCCTGTTGCTGTAGCCATATT |  |
